# Assessment of the Technical Condition of Timber Structural Elements Using Sclerometric Tests

**DOI:** 10.3390/ma16186152

**Published:** 2023-09-10

**Authors:** Justyna Jaskowska-Lemańska, Daniel Wałach

**Affiliations:** Faculty of Civil Engineering and Resource Management, AGH University of Science and Technology, Adama Mickiewicza Ave. 30, 30-059 Kraków, Poland; walach@agh.edu.pl

**Keywords:** timber structures, semi-destructive tests, sclerometric tests, technical condition assessment, dynamic hardness, wood surface, mechanical properties

## Abstract

The technical assessment of wooden elements is the primary step in their repair and reinforcement design. Normative requirements currently mandate additional tests, including semi-destructive ones, beyond traditional visual assessment. Despite the growing feasibility of semi-destructive tests for qualitative assessments, there remains a paucity of data enabling quantitative assessments. This study investigated the hardness of structural timber, specifically pine, spruce, and fir, from Central Europe using sclerometric methods. The outcomes of these tests were compared with those of conventional destructive tests and correlational relationships were established. A strong correlation was found between the sclerometric tests and density (r = 0.62 ÷ 0.82), while a range of strong to moderate correlations was found (r = 0.40 ÷ 0.70) for mechanical characteristics (bending and compressive strength). The correlation strength varied among different wood species, with the strongest for pine and the weakest for spruce. All established relationships were compiled into 40 functions to facilitate their future utilization in quantitative assessments during the technical evaluation of wooden objects. The study also examined the influence of wood defects on the derived correlations by considering the knot index. Sclerometric methods accurately reflect the physico-mechanical properties of elements with a small or medium defect content. However, for wood with a high proportion of defects (knots), the correlations are very weak (r = 0.23 ÷ 0.52, including statistically insignificant results). This research offers new insights into the potential of semi-destructive methods in the structural evaluation of wooden elements, highlighting the need to account for wood species and defect content.

## 1. Introduction

Structural wooden elements, particularly those in heritage buildings, often require more frequent repairs and reinforcements than those made from other materials. This need is especially evident when the elements are exposed to atmospheric and biological factors or improper use [1]. To adequately execute repairs and reinforcements, it is necessary to determine the current load-bearing capacity of these timber elements, most often floors and roof trusses [2,3]. The load-bearing capacity of any construction is dependent on its geometrical and physical–mechanical characteristics. In the case of existing wooden structures, natural defects and damage resulting from long-term use are additional significant factors [4,5].

The physical–mechanical properties of wood in existing constructions can be determined through destructive (DT), non-destructive (NDT), and semi-destructive tests (SDT) [6,7,8]. Destructive tests provide the most reliable results but must be limited, particularly in heritage buildings, due to their destructive nature. Within the semi-destructive testing realm, scraping resistance tests [9], tear tests [10], and hardness tests [11] are noted. In recent years, a significant increase in the use of scraping resistance tests and hardness tests has been observed, correlated with the increased availability of the necessary testing equipment. Scraping resistance tests are commonly used in material quality assessment but rarely proposed for quantitative assessments. In contrast, hardness tests, due to their localized nature, are most often used for quantitative evaluations but should not be applied as standalone research methods. It is crucial to note that the current testing procedures for the technical assessment of wooden elements highlight the necessity of employing various methods jointly—particularly, visual grading combined with various semi-destructive tests [12,13,14]. Despite these normative indications, there is a lack of correlational relationships in the literature between SDT results and the physical–mechanical properties of different wood species from various regions of the world.

In this study, the focus is placed on hardness tests. In the context of wood testing, these tests can be classified as semi-destructive since they leave minor traces (indentations or holes) on the tested element upon completion. Hardness measurements commenced with the development of the Brinell and Rockwell methods in the early 20th century. Since then, they have evolved to meet the need for measurements using different force levels and materials other than those originally intended. Hardness tests are frequently used due to their ease and speed of execution. Unfortunately, when it comes to wooden elements, these tests are more challenging to conduct. This is related to the increased number of factors influencing the physical–mechanical characteristics of wood, and, consequently, the test results [15].

In practice, static methods such as those by Janka, Brinell, and Monnin are used in wood testing. These methods utilize the relationship of deformation under constant load and are applicable in testing finishing elements [15]. Due to the type of instrumentation and the availability of testing surfaces, these methods are not typically used for structural elements. Piazza and Turrini proposed a modification of these methods for assessing the technical condition of structural elements, deriving a correlational relationship between the static force required to embed a pin with a diameter of 10 mm to a depth of 5 mm and the longitudinal elasticity modulus. The relationship was developed for fir, larch, and chestnut at 15% humidity, incorporating a macroscopic assessment by applying an appropriate correction factor [16,17]. Obstacles to the application of the above method include the limited number of wood species for which correlational curves have been created. Furthermore, the equipment is not commercially available and requires anchoring in the tested element, significantly increasing the range of damage incurred during the test.

The second group of hardness tests used in assessing the condition of wooden structural elements are tests based on the dynamic penetration of the material with a thin indenter (often referred to as sclerometric tests). These tests involve measuring the penetration depth of a steel needle/pin introduced into the material using a mechanical hammer with a constant impact energy. The depth of the indentation allows for the qualitative assessment of the surface layers of the element and the identification of areas with lower properties [7]. Holes left after dynamic testing do not affect the load-bearing capacity of wooden elements and, depending on the chosen pin/needle, have a minimal or small impact on their aesthetics [18]. The advantages of using dynamic hardness tests include a short testing time and instant measurement results. The primary limitations of using wood hardness measurement methods for a quantitative assessment of technical conditions include the lack of correlation curves for selected species of wood from a given region, as well as the local nature of the test. Currently, there are two types of commercially available devices for the dynamic hardness testing of wood: Pilodyn and WoodTester. Pilodyn was initially used for testing wooden telecommunication poles with a circular cross-section and standing trees [19]. Despite the availability of devices with different energies, the model with an energy of 6 J is the most popular. WoodTester was created based on the Schmidt sclerometer for concrete testing (similar energy and construction); the most likely prototype was from the studies conducted by Giuriani and Gubana using this device [20]. In this study, the WoodTester device from Novatest was used due to fewer variables (one impact energy—2.4 J—and one needle diameter) than in the case of the Pilodyn device, and the increasingly common availability on the market of devices with these same parameters (impact energy and type of indenter).

Establishing the relationships between the physical–mechanical properties of wood and the results of destructive and semi-destructive tests may allow for the application of diagnostic devices to determine these features without carrying out costly and destructive procedures on the structures. Due to significant variations in the physical–mechanical characteristics of different wood species, there is a pressing need for studies that consider the wood’s origin. Currently, Central European wood species have not received sufficient research attention. This work also addresses an additional aspect that has been largely overlooked in the existing literature: the incorporation of visual assessments into the context of correlation relationships. The derivation of correlation relationships for selected wood species and the proposal of their potential application in determining characteristic values will enable more effective repairs and reinforcements of wood structures to be carried out in the future.

## 2. Materials and Methods

**Figure 1:**
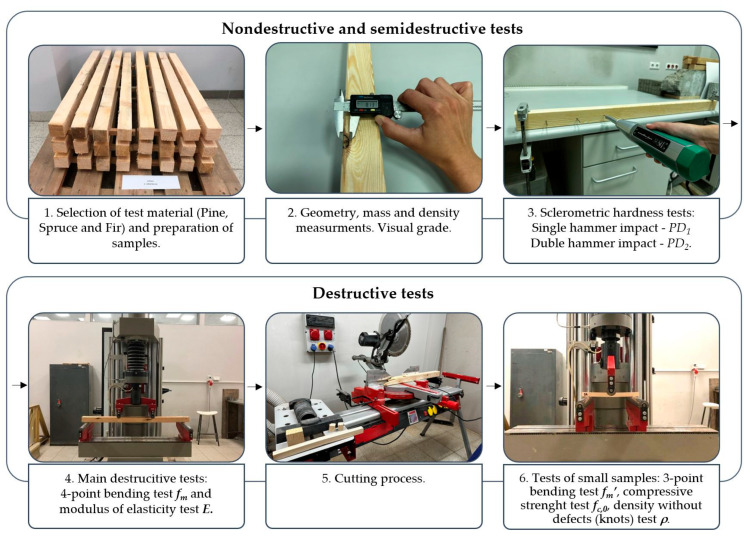
Research procedure scheme.

The analysis included pine, spruce, and fir beam elements with dimensions of approximately 50 mm × 50 mm × 1000 mm. To obtain a sample diverse in terms of density, wood from many different habitats in Central Europe was used. All these species can be classified as soft coniferous wood—these are currently the most commonly used species in construction in this region. The beams were seasoned for three months in a room where the average temperature was approximately +21 °C and the humidity was between 50% and 60%. The humidity of the beams at the time of testing was between 11.1% and 11.7%. Figure 1 schematically presents the conducted research procedure.

The precise arrangement of points for individual tests and the locations for the small sample collection were each time adapted to the given piece of lumber so that the marking was carried out without the influence of existing defects in the element (particularly knots) and while excluding points that were damaged during the SDT or DT tests.

### 2.1. Sample Selection and Non-Destructive Testing

As part of the non-destructive testing, the weight of the sample, its geometric characteristics, and assignment to a quality class were measured (the type, dimensions, and degree of visible wood defects are taken into account when determining the quality class). The sorting was based solely on the knot area ratio (KAR)—this is an index that characterizes the proportion of knots on the cross-sectional surface of the timber, with two values of the index distinguished: the total knot area ratio (TKAR), related to the entire cross-section, and the marginal knot area ratio (MKAR), related to the so-called marginal zone with a height of ¼ of the cross-sectional height, where the highest concentration of knots occurs. The knot area ratio is assessed in the cross-section with the highest concentration of defects, regardless of the distance of this concentration from the end of the element. Further details of the method of assessing the quality class and the KAR index are presented in the works of [21,22], among others. The assignment of a given piece of timber to a certain quality class was made based on the standard PN-D-9402:2013 [23]—which is based on European guidelines contained in the standard EN 14081-1:2007 [24]. Three quality classes were taken into account: KW—best quality, KS—medium quality, and KG—inferior quality. If defects other than knots were present, the element was discarded from the test sample. Samples with a very high knot area ratio (TKAR > 1/2; MKAR > 1/2) were also excluded from the tests. The sample selection was conducted in such a way as to obtain an equal share of samples from each sorting class.

The features of the obtained test sample are presented in Table 1.

The obtained test results do not deviate from the wood characteristics presented in the literature from the Central European region which was used to determine that the obtained sample is reliable.

### 2.2. Hardness Testing

**Figure 2:**
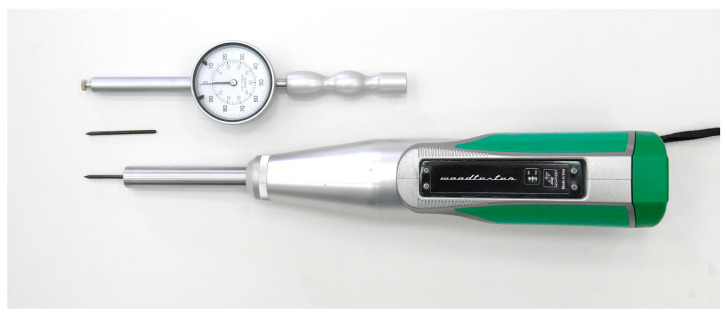
Woodtester Novatest set.

Sclerometric tests were performed using a device (Woodtester Novatest Ancona, Italy) with an impact energy of 2.4 J equipped with steel needles of 60 HR hardness, a diameter of 2.5 mm with a conical tip inclined at 35° and a length of 50 mm, and a dial gauge enabling measurement with an accuracy of 0.01 mm. The device was calibrated on a control anvil with a hardness of >52 HRC. The device manufacturer suggests five hits in one test point, however, for soft coniferous wood, five hits cause a needle indentation larger than the device’s measurement scale. The markings were made for a horizontal device layout for a single (PD_1_) and double (PD_2_) indentation/hit (i.e., a double hit on the needle without prior removal). The measured value was the remaining portion of the needle that was not indented. The difference between the length of the needle and the measured size was treated as the measurement result. After taking readings after the first and second indentation, the needles were removed from the element using hand tools. The measurement set is shown in Figure 2.

**Figure 3:**
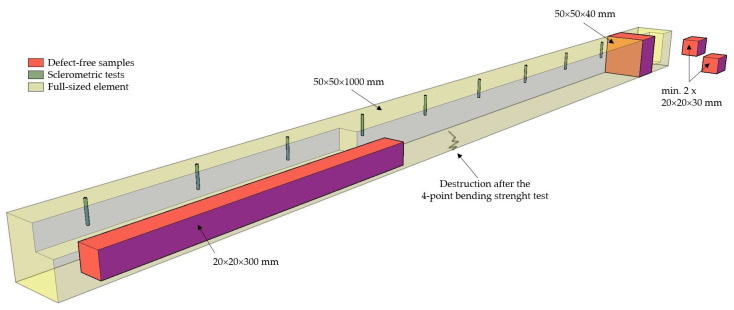
Schematic division of the test element and the location of individual test samples.

Each element had nine measurement points, and the result of the test was treated as the arithmetic mean of these measurements. By assumption, sclerometric tests were conducted along the longitudinal axis of the element, and the points were located approximately every 10 cm and no less than 10 cm from its face. Tests were conducted perpendicular to annual growths outside the knot areas. The scheme of distribution of research points is shown in Figure 3.

### 2.3. Destructive Testing

Destructive tests were carried out in two stages: in the first stage, the full-sized element was tested for its four-point bending strength and longitudinal modulus of elasticity, in the second stage, after cutting the previously destroyed element, bending and compression strength tests were carried out on small, flawless samples. In addition, the density of the defect-free sample was also determined [25]. An example of an element cutting scheme after destruction is shown in Figure 3.

Destructive tests from the first stage were performed in relation to standard procedures. Bending strength (f_m_) was determined by a four-point method with a simultaneous determination of the bending elasticity modulus (E) [26] on a technical scale sample measuring 50 mm × 50 mm × 1000 mm with a load speed corresponding to 0.003 h mm/s (where h is the height of the sample). The support distance was 900 mm and the distance of concentrated forces from the supports was 300 mm. Displacement measurement was performed in the longitudinal axis of the element using an external sensor. A linear segment of the force–displacement relationship was used to calculate the longitudinal modulus of elasticity.

Destructive tests from the second stage were performed in relation to standard procedures. Bending strength (f_m_’) was determined by a three-point method on a defect-free sample measuring 20 mm × 20 mm × 300 mm [27] and compression strength along the fibers (f_c_) was determined on a defect-free sample measuring 20 mm × 20 mm × 30 mm [28]. For each beam, at least two samples were taken for compression strength testing, and the average value was considered as the result.

## 3. Results

The results are presented based on wood species and the selected physical–mechanical characteristics under study. The paper explores two types of dependencies. In the first part, dependencies are discussed without considering the visual grade, meaning that they are analyzed irrespective of the number of defects present in the element. In the second part, dependencies are taken into account with a visual grade of the presented defects. This involves analyzing the dependencies within groups that are divided according to the number of defects present in the element.

### 3.1. Correlation Dependencies without Considering Visual Grade

The results are presented divided by the type of correlation between the physico-mechanical characteristic and the sclerometric test results. For individual species of wood, the correlations are presented in the form of scatter plots with a trend line (solid line), confidence ranges (dashed lines), and the correlation coefficient r (Pearson’s linear correlation). For the collective analysis, where all results were taken without dividing by species of wood, the trend line was determined in the form of a quadratic function, and the determination coefficient R^2^ was given. All the results analyzed were statistically significant for *α* = 0.05. All derived correlation functions are compiled in Appendix A.

**Figure 4:**
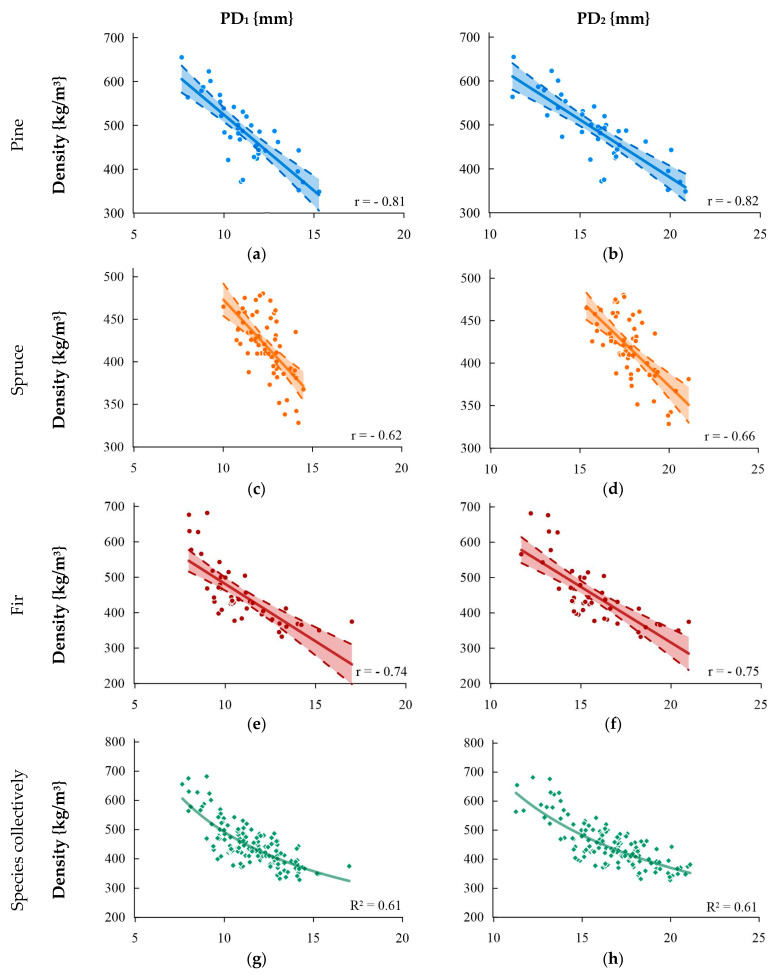
Relationship between volumetric density and the result of the sclerometric test PD: (**a**) pine—single impact, (**b**) pine—double impact, (**c**) spruce—single impact, (**d**) spruce—double impact, (**e**) fir—single impact, (**f**) fir—double impact, (**g**) species collectively—single impact, and (**h**) species collectively—double impact.

Figure 4 shows the correlation between the density and the sclerometric test for single (PD_1_) and double indentations (PD_2_). Very strong correlations were obtained for pine wood and collective analysis. The lowest correlation was obtained for spruce wood which can be described as moderate.

**Figure 5:**
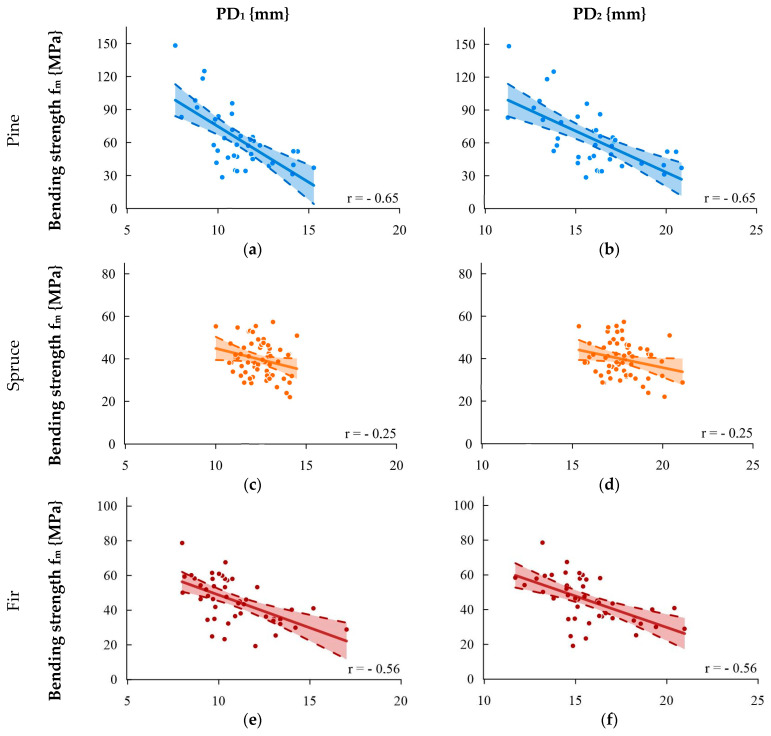

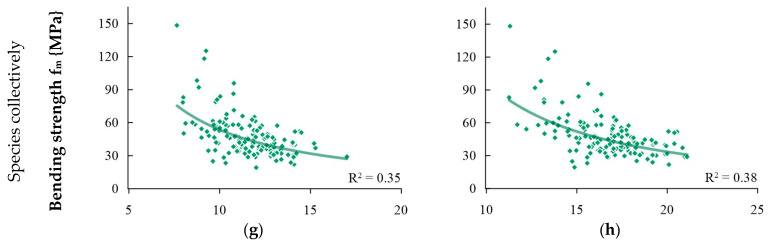
Relationship between the bending strength and result of the sclerometric test PD: (**a**) pine—single impact, (**b**) pine—double impact, (**c**) spruce—single impact, (**d**) spruce—double impact, (**e**) fir—single impact, (**f**) fir—double impact, (**g**) species collectively—single impact, and (**h**) species collectively—double impact.

Figure 5 shows the correlation between the bending strength (determined by the element on a technical scale) and the sclerometric test for single (PD_1_) and double indentations (PD_2_). Similar to the correlation with density, the strongest correlation was obtained for pine wood and collective analysis, with a slightly lower correlation for fir wood; a very low correlation was obtained for spruce wood. During the study, correlations between the bending strength determined in the small defect-free sample test were also analyzed. The correlations obtained in this test were always higher than for tests of samples on a technical scale (with defects). This results directly from the large and somewhat unpredictable influence of wood defects and their distribution on the bending strength of the element. For spruce beams on a technical scale, a weak correlation (r = 0.25) was obtained. While analyzing defect-free samples taken from the same beams, the correlation coefficient for the penetration depth–bending strength relationship increased to r = 0.50 (PD_1_) and r = 0.51 (PD_2_). A similar increase in the correlation coefficient was observed for fir wood, while for pine wood, the registered increase was not as significant (increase in r by 0.05). 

**Figure 6:**
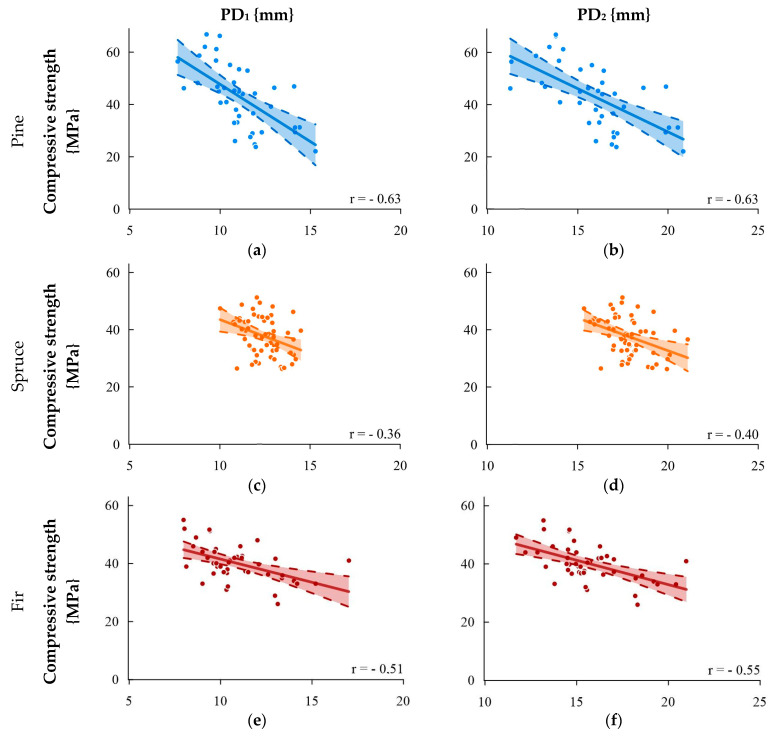

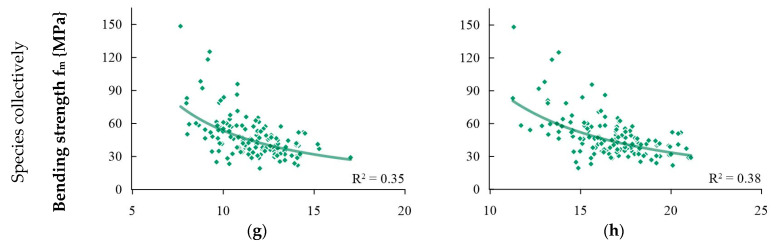
Relationship between the compressive strength and result of the sclerometric test PD: (**a**) pine—single impact, (**b**) pine—double impact, (**c**) spruce—single impact, (**d**) spruce—double impact, (**e**) fir—single impact, (**f**) fir—double impact, (**g**) species collectively—single impact, and (**h**) species collectively—double impact.

Figure 6 shows the correlation between the compression strength and the sclerometric test for single (PD_1_) and double indentations (PD_2_). The strongest correlations were obtained for pine and fir wood. Correlations for spruce wood were low. In the collective analysis, the determination coefficient significantly differed for single and double indentations.

**Figure 7:**
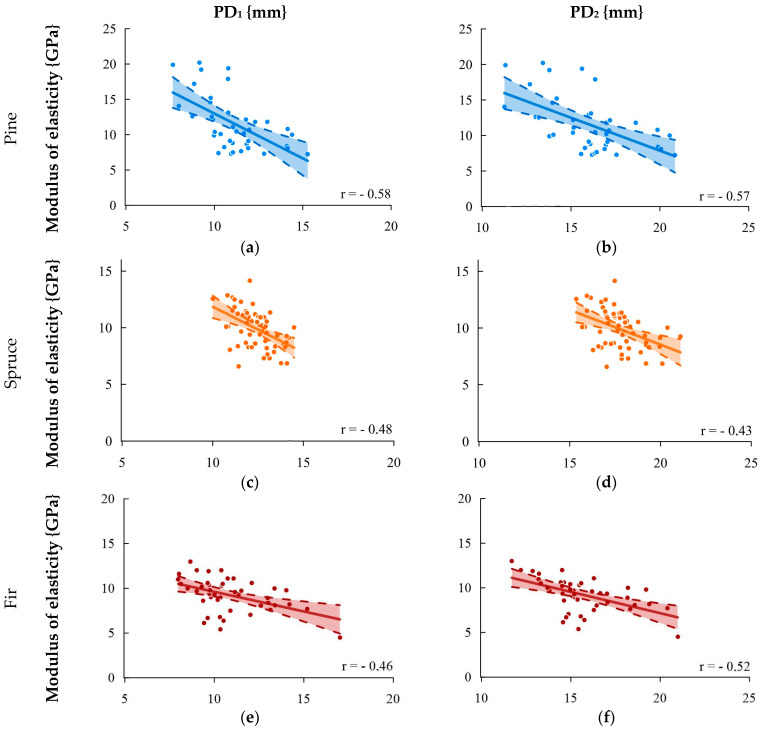

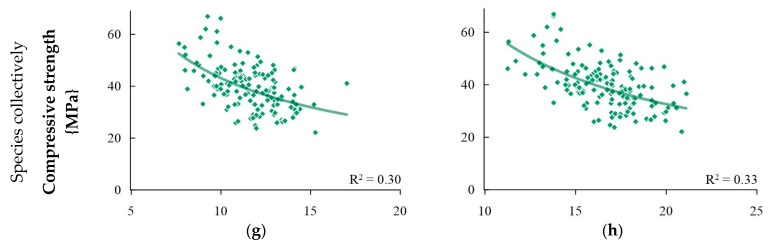
Relationship between the modulus of elasticity and result of the sclerometric test PD: (**a**) pine—single impact, (**b**) pine—double impact, (**c**) spruce—single impact, (**d**) spruce—double impact, (**e**) fir—single impact, (**f**) fir—double impact, (**g**) species collectively—single impact, and (**h**) species collectively—double impact.

In Figure 7, the correlation between the longitudinal modulus of elasticity and the sclerometric test for single (PD_1_) and double indentations (PD_2_) is presented. Generally, the correlation of needle indentation for all species was lower than with other physico-mechanical characteristics. Nevertheless, as in previous correlations, the strongest correlations were obtained for pine wood and the lowest for spruce wood.

### 3.2. Correlation Dependencies Considering Visual Grade

**Figure 8:**
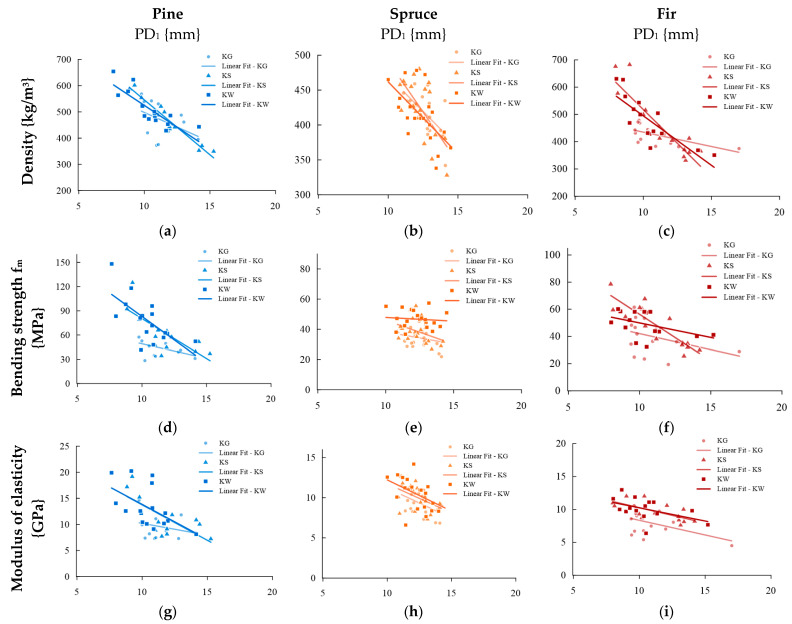
Example dependencies obtained for individual quality classes. Density: (**a**) pine—single impact, (**b**) spruce—single impact, (**c**) fir—single impact; Bending strength: (**d**) pine—single impact, (**e**) spruce—single impact, (**f**) fir—single impact; Modulus of elasticity: (**g**) pine—single impact, (**h**) spruce—single impact, (**i**) fir—single impact.

In dividing into quality classes, the correlation between sclerometric tests and volumetric density, bending strength on a technical scale, and the modulus of elasticity was analyzed. Figure 8 exemplifies the relationships divided into sorting classes. All derived correlation functions are compiled in Appendix B.

All obtained correlation coefficient r results for individual quality classes are compiled in Table 2.

For pine wood within the average (KS) and selected (KW) classes, higher correlation coefficients were usually obtained than for the data analyzed collectively. Within the lower class (KG), this value is lower. For spruce wood, the correlation coefficient between the depth of needle penetration and the density of the sawn timber is comparable in the collective analysis and the analysis in separate sorting classes. In all classes, comparable or higher correlation coefficients were usually obtained with the bending strength and modulus of elasticity than for the collective analysis; the exception is the correlation with bending strength in the selected class, which is significantly lower. For fir wood, within the lower class, a lower correlation coefficient was obtained between the depth of needle penetration and the density of the sawn timber, and the bending strength and a comparable correlation value for the modulus of elasticity. In the middle class, all obtained correlations were higher than in the case of the collective analysis and most of them were very strong. Within the selected class, a higher correlation coefficient was obtained than in the case of the collective analysis between the depth of needle penetration and the density of the sawn timber (very strong correlation). The other values are comparable to the collective analysis.

## 4. Discussion

The obtained results indicate the existence of relationships between physical and mechanical properties and the results of sclerometric tests. The strength of these dependencies is variable. These dependencies also differ within individual types of wood. All of the obtained correlation coefficient r and determination coefficient R^2^ results for tested wood samples are compiled in Table 3.

The strongest correlations were obtained for pine wood and then fir wood. For spruce wood, despite a larger research sample compared to other species, correlations at a similar level were not obtained; this is probably due to the smaller range of density of the tested samples. For research on this species, it was only possible to obtain material with densities in the range of 350 to 480 kg/m^3^, while the literature states that spruce wood from habitats in Central Europe reaches densities in the range of 330 to 680 kg/m^3^ [29,30]. In other cases, it was possible to obtain material with a larger density range (370 to 840 kg/m^3^ for pine and 345 to 715 kg/m^3^ for fir) similar to the ranges presented in the literature. Due to the fact that the values of the correlation and determination coefficients for single and double needle penetration were often very similar, they were treated together in the rest of the discussion.

For each type of wood, the highest correlations of the depth of the sclerometer needle penetration with volumetric density were obtained (r in the range of 0.62 ÷ 0.82). These are higher correlation coefficient values than those presented by Kloiber et al. [31] who also studied these relationships for pine, spruce, and fir wood, but used the Pilodyn 6 J device and obtained a correlation coefficient in the range of 0.54 ÷ 0.68. However, it should be emphasized that these tests were performed on samples taken from one log. A similar to Kloiber et al. [31] correlation coefficient value r = 0.54 was also obtained by Faggiano et al. [32] for historical chestnut wood (using the Woodtester device). In turn, Giefing and Ro-manowska [33], Lourenco, Feio, and Machado [34], and Henriques et al. [35] obtained stronger correlations (r = 0.86 to 0.95) for the relationship between the penetration depth and sawn timber density than presented in this work.

**Figure 9:**
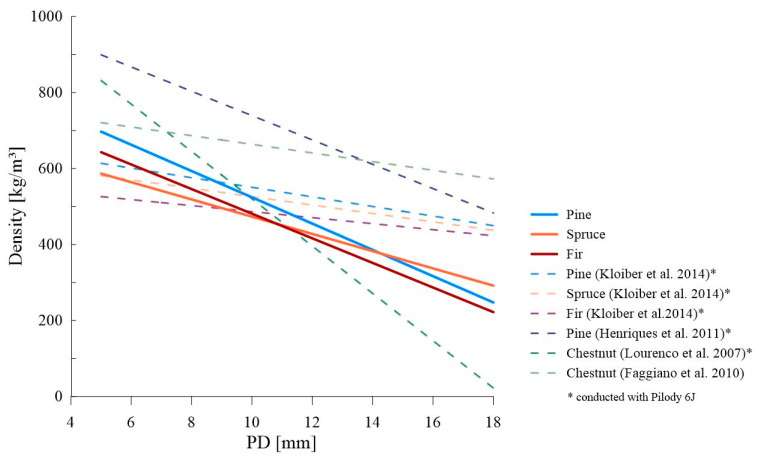
Comparison of the literature data with the derived linear dependencies (PD_1_—*ρ*_0_) [31,32,34,35].

Figure 9 presents a comparison of the linear relationships obtained from our research (for a single impact) against the background of the works cited above with the note that some of the cited studies were performed using the Pilodyn 6 J device. Comparable to that presented in these studies, the direction of the slope of the curve (slope coefficient) was obtained by Henriques et al. [35], although the correlation value itself was definitely different (a much higher constant term in the regression equation). On the other hand, for penetration depths in the range of about 8 to 10 mm, the results obtained partially coincide with the dependencies derived by Kloiber et al. [31], however, their dependencies had a much lower slope coefficient. It is also worth noting the differences between the studies for pine wood performed by Henriques et al. [35] and Kloiber et al. [31] whose correlations were quite divergent, despite using the same device, especially for small penetration depths. This is probably due to the little diversified sample in the work [31] and also the different growth conditions of pine in the areas of conducted research.

The second correlation found in the literature is the relationship between the depth of the sclerometer needle penetration and the compressive strength. These correlations are often weaker than those for density. In the studies by Kloiber, Tippner, and Hrivnák [31], a decrease in the value of r by 0.15 for spruce and fir wood was observed, along with a slight increase for pine wood. Meanwhile, Henriques et al. [35] obtained a correlation lower by 0.11 compared to the correlation with density. Lourenco, Feio, and Machado [34] obtained results below statistical significance, whereas, in the same studies for the correlation with density, the correlation coefficient r was equal to 0.87. On the other hand, Faggiano et al. [32] obtained a strong correlation for historical chestnut wood using the Woodtester device, which was greater by 0.21 than in the case of the same tests for density. This variation may result from different compressive strength testing methods (sample size) and a more challenging interpretation of the destructive loads for small compression samples. For the analyzed results of our research, a lower correlation coefficient value was obtained in every case than for the correlation with the density of the sawn timber—this decrease averaged 0.2.

**Figure 10:**
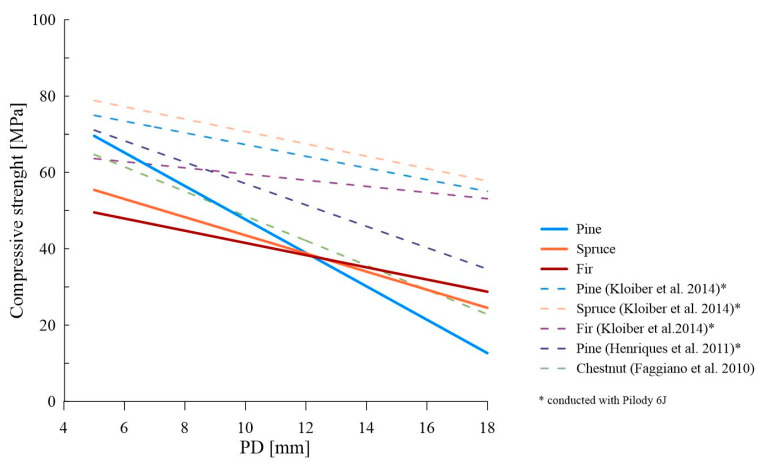
Comparison of the literature data with the derived linear dependencies (PD_1_—f_c,0_) [31,32,35].

Figure 10 presents a comparison of the derived linear dependencies from our research (for a single impact) against previously referenced works. For pine wood, the largest slope coefficient is evident, closest to the results of Henriques et al. [35] and Faggiano et al. [32]. The results obtained for spruce wood in terms of the slope coefficient are closest to the results obtained by Kloiber et al. for pine and spruce [31]. Also, the results obtained for fir wood in this range can be compared to the results obtained for fir from the work of Kloiber et al. For all the correlations obtained in this study, the y-intercept was generally lower than that presented in the literature data, which may result from the research methodology (the compressive strength in our studies was determined on small samples without defects—20 mm × 20 mm × 30 mm).

Faggiano et al. [32] also attempted to correlate the depth of the sclerometer needle penetration to bending strength, eventually obtaining a moderate correlation (*r* = 0.44). This low correlation value may be associated with the fact that the technical scale bending strength tests were only conducted for 10 historical beams with little density differentiation. Currently, in the literature, no more results have been encountered for this correlation, which may be due to the fact that preparing samples for tests is more troublesome than in the case of density and compressive strength testing, as well as the theoretically possible conversion of this feature from the above values, i.e., density or compressive strength.

**Figure 11:**
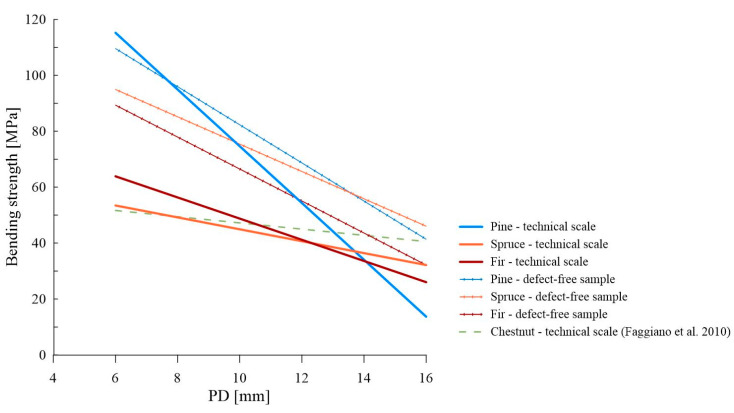
Comparison of the literature data with the derived linear dependencies (PD_1_—f_m_ and PD_1_—f_m_’) [32].

Figure 11 presents a comparison of the plotted derived linear dependencies from our research (both for the correlation to bending strength determined on a technical scale sample and for a small sample without defects) and the relationship derived by Faggiano et al. for chestnut wood. The literary relationship significantly deviates from the obtained results, which may be related to a completely different species of wood (deciduous non-heartwood wood of a diffuse-porous structure). The derived dependencies for tests performed on a technical scale differed significantly from each other within individual species, not only in terms of the y-intercept but also the slope coefficient. On the other hand, dependencies for tests performed on small samples without defects were decidedly more similar to each other, both in terms of the y-intercept and the slope coefficient. The arrangement of these three relationships in this case, almost throughout, was consistent with the literature data regarding mechanical properties (generally, the highest bending strength is characteristic of pine wood, intermediate for spruce wood, and the lowest for fir wood).

The next characteristic analyzed was the local modulus of elasticity along the fibers which was determined on beams bent on a technical scale. In the literature, references to the dynamic modulus of elasticity determined from non-destructive tests are the most often found, which may be due to the speed of performing such a determination compared to destructive tests [22].

Schlerometric studies were conducted outside the area affected by the occurrence of knots; however, due to the significant impact of the occurrence of wood defects on the decrease in the mechanical properties of the material, correlations between schlerometric studies and physico-mechanical characteristics (density of the cut-out, bending strength on a technical scale, and the local modulus of elasticity along the fibers) were also considered and divided into sorting classes (determined due to the knottiness of the elements). The literature describes a decrease in mechanical properties with the occurrence of knots from 6% to 35% [29,36,37]. The extent of changes caused by wood defects depends on the size of the knots, their location, shape, and how much fiber inclination they cause within a given element. Within the lower sorting class, for virtually every species of wood and every type of study, correlations were obtained that were lower or comparable to the results treated collectively. Within the average sorting class, an increase in the correlation coefficient was the most often observed. However, for the selected class, we did not observe a clear effect—for fir and pine, it was most often a comparable or higher value, while for spruce it was most often a comparable or slightly lower value. Globally, the highest correlation coefficients were obtained by considering only samples of the middle and selected classes. This explains the higher correlation coefficients presented in the literature for studies that were conducted exclusively on samples without defects or with minimal participation of them. In this work, it was decided to create relationships from all classes together due to the possibility of the presence of lower-class elements in structures as well.

**Figure 12:**
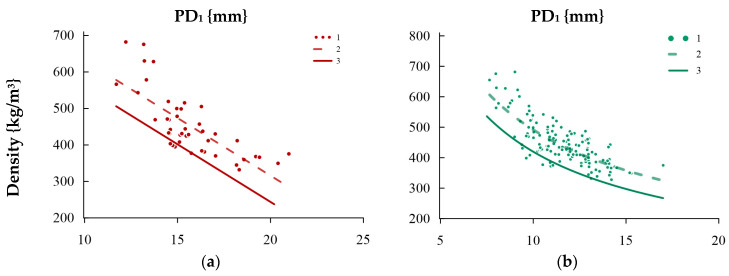
Example dependency relationships between the volumetric density and result of the PD_1_ sclerometric test corresponding to the characteristic values for (**a**) fir and (**b**) the species collectively. Legend: 1—test results, 2—correlation relationship, and 3—relationship corresponding to the characteristic value.

The relationships presented above fit into the literature values and allow for the statement that it is possible to use schlerometric tests as a supplement to the visual grade for the evaluation of the physico-mechanical characteristics of wooden constructions in Central Europe. For engineering applications, it is suggested to use linear relationships that have been brought to a safe level (the transformation of functions corresponding to the 5% safety quantile—i.e., no more than 5% of results are below a given function). Figure 12 shows example charts of the relationships with individual test results and the primary and transformed linear relationship. These correlations in the form of functions are included in Appendix C. However, it should be noted that these relationships should not be extrapolated beyond the given range of applicability and used with great caution for wood of the same species (or the same type) originating from significantly different habitat conditions.

## 5. Conclusions

Wood, as a natural anisotropic material commonly used in construction, is a very interesting research object. Both within classic destructive tests and semi-destructive tests, researchers face many problems resulting from its structure. The presence of natural defects such as knots, fiber twists, or cracks affects the obtained test results. Moreover, during the tests, a number of external factors such as wood moisture, test temperature, or the way the sample is loaded relative to the annual increments, which significantly affect the results of these tests (DT, NDT, and SDT), should be taken into account [38,39,40,41,42]. Nevertheless, it seems reasonable to conduct research aimed at developing NDT and SDT techniques for historical or heritage wooden objects as they can be a good source of information about wooden structures and their properties and thus can be a basis for designing the repairs and reinforcements of these objects.

In the study, correlation relationships were determined between the physico-mechanical characteristics and results of sclerometric tests for the three most commonly used types of wood in Central Europe: pine, spruce, and fir. The strength of the correlations obtained between the DT and SDT studies was varied, which is a natural phenomenon for wood studies. Increasing the strength of these correlations and expanding the scope of their applicability can be achieved by increasing the sample size with studies for extreme density ranges for the selected types of wood. Due to the nature of the material, i.e., wood, whose characteristics strongly depend on its origin conditions, the obtained results are not considered as a closed set but rather as a base which should be systematically supplemented with further measurements.

For practical applications, correlation relationships corresponding to characteristic values in the sense of the PN-EN 384: 2011 standard were derived [25], i.e., the 5% quantile of the probability distribution of individual wood properties, separately for each analyzed species and as a cumulative result for all analyzed species. These proposals may form the basis for supplementing the EN 17121: 2019 [12] standard in terms of appendices concerning the rules for determining the physico-mechanical properties of Central European construction wood using sclerometric tests.

Further work should focus not only on expanding the correlation relationships for additional types of wood and wider ranges of its density but also on the influence of individual factors on the results of these studies. In the literature, references can already be found regarding the impact of various factors on the results of the NDT and SDT tests; however, in most cases, these are not quantitative but only qualitative data. The most important factors that have not yet been fully identified include the impact of temperature, stress, and age and biological corrosion. This offers fertile ground for future investigations, as understanding these influences can lead to advancements not only in wood science but also in related fields, including materials engineering, heritage preservation, and environmental science. By bridging the gap between wood’s intricate properties and its practical applications, our research contributes to a deeper understanding of this natural resource and its potential impact on various scientific disciplines.

## Figures and Tables

**Table 1:** Physical and mechanical properties of individual test samples.

	Pine	Spruce	Fir
Sample size {pcs.}	45	60	45
Sample density {kg/m^3^} min/**mean**/max *standard deviation*	348.5/**485.8**/655.2 *74.2*	328.2/**416.9**/480.5 *36.6*	332.4/**452.9**/682.1 *86.1*
Moisture content {%} min/**mean**/max *standard deviation*	10.2/**11.1**/12.3 *0.72*	11.3/**11.7**/12.2 *0.20*	10.0/**10.6**/11.2 *0.35*
Bending strength f_m_{MPa} min/**mean**/max *standard deviation*	28.5/**63.4**/148.2 *27.1*	22.0/**39.5**/57.3 *8.5*	19.2/**45.4**/78.6 *13.1*
Modulus of elasticity {GPa} min/**mean**/max *standard deviation*	7.3/**11.6**/20.2 *3.8*	6.6/**9.9**/14.2 *1.7*	4.5/**9.2**/13.0 *1.9*
Compressive strength f_c,0_ {MPa} min/**mean**/max *standard deviation*	22.1/**42.9**/66.8 *12.2*	26.3/**37.6**/51.3 *6.7*	26.1/**40.2**/55.4 *6.2*

**Table 2:** Obtained correlation coefficient r values for the selected relationships considered in a given quality class.

		Pine		Spruce		Fir	
		PD_1_	PD_2_	PD_1_	PD_2_	PD_1_	PD_2_
		{mm}	{mm}	{mm}	{mm}	{mm}	{mm}
KW	ρ {kg/m^3^}	−0.84	−0.81	−0.62	−0.72	−0.81	0.79
	f_m_ {MPa}	−0.68	−0.66	−0.10 ^1^	−0.15 ^1^	−0.44	−0.51
	E {GPa}	−0.49	−0.62	−0.46	−0.43 ^1^	−0.48	−0.59
KS	ρ {kg/m^3^}	−0.97	−0.96	−0.66	−0.64	−0.86	0.84
	f_m_ {MPa}	−0.71	−0.71	−0.39	−0.45	−0.86	0.74
	E {GPa}	−0.76	−0.74	−0.45	−0.41	−0.81	0.57
KG	ρ {kg/m^3^}	−0.43 ^1^	−0.57	−0.62	−0.63	−0.60	−0.54 ^1^
	f_m_ {MPa}	−0.39 ^1^	−0.46 ^1^	−0.23 ^1^	−0.26 ^1^	−0.37 ^1^	−0.31 ^1^
	E {GPa}	−0.25 ^1^	−0.32 ^1^	−0.49	−0.52	−0.47 ^1^	−0.52 ^1^

^1^ *p*-value > 0.05—no grounds for rejecting the null hypothesis.

**Table 3:** Obtained correlation coefficient r and determination coefficient R2 values for the selected relationships.

			Pine r		Spruce r		Fir r		Species Collectively R^2^	
			PD_1_	PD_2_	PD_1_	PD_2_	PD_1_	PD_2_	PD_1_	PD_2_
Density	ρ	{kg/m^3^}	−0.81	−0.82	−0.62	−0.66	−0.74	−0.75	0.61	0.61
Bending strength	f_m_	{MPa}	−0.65	−0.65	−0.25	−0.25	−056	−0.56	0.35	0.38
Bending strength ^1^	f_m_’	{MPa}	−0.70	−0.69	−0.50	−0.51	−0.72	−0.75	0.41	0.43
Compressive strength	f_c,0_	{MPa}	−0.63	−0.63	−0.36	−0.40	−0.51	−0.55	0.30	0.33
Modulus of elasticity	E	{GPa}	−0.58	−0.57	−0.48	−0.43	−0.46	−0.52	0.21	0.24

^1^ small defect-free sample.

## Data Availability

Data are contained within the article and Appendix A, Appendix B and Appendix C.

## Appendix A

**Table A1:** Obtained correlation functions.

			PD_1_	PD_2_	
Pine	Scope		7.5 ÷ 15.5	11.0 ÷ 21.0	{mm}
	Density	ρ	= −34.593·PD_1_ + 870.06	= −26.299·PD_2_ + 906.23	{kg/m^3^}
	Bending strength	f_m_	= −10.152 PD_1_ + 176.14	= −7.5324·PD_2_ + 183.79	{MPa}
	Bending strength	f_m_’	= −6.8236 PD_1_ + 150.54	= −5.0348·PD_2_ + 155.24	{MPa}
	Compressive strength	f_c,0_	= −4.3784 PD_1_ + 91.476	= −3.3075·PD_2_ + 95.719	{MPa}
	Modulus of elasticity	E	= −1.2683 PD_1_ + 25.671	= −0.926·PD_2_ + 26.386	{GPa}
Spruce	Scope		10.0 ÷ 15.0	15.0 ÷ 21.5	{mm}
	Density	ρ	= −22.687·PD_1_ + 700.07	= −20.227·PD_2_ + 777.65	{kg/m^3^}
	Bending strength	f_m_	= −2.1265·PD_1_ + 66.188	= −1.7908·PD_2_ + 71.594	{MPa}
	Bending strength	f_m_’	= −4.8925·PD_1_ + 124.3	= −4.1546·PD_2_ + 137.35	{MPa}
	Compressive strength	f_c,0_	= −2.3761 PD_1_ + 67.301	= −2.2765·PD_2_ + 78.234	{MPa}
	Modulus of elasticity	E	= −0.8063·PD_1_ + 19.915	= −0.614·PD_2_ + 20.811	{GPa}
Fir	Scope		7.5 ÷ 17.5	11.0 ÷ 21.0	{mm}
	Density	ρ	= −32.383· + 804.88	= −31.568·PD_2_ + 947.23	{kg/m^3^}
	Bending strength	f_m_	= −3.7841·PD_1_ + 86.574	= −3.6131·PD_2_ + 102.02	{MPa}
	Bending strength	f_m_’	= −5.7204·PD_1_ + 123.67	= −5.8014·PD_2_ + 153.3	{MPa}
	Compressive strength	f_c,0_	= −1.5976·PD_1_ + 57.519	= −1.6767·PD_2_ + 66.411	{MPa}
	Modulus of elasticity	E	= −0.44·PD_1_ + 14.017	= −0.4768·PD_2_ + 16.701	{GPa}
Species collectively	Scope		7.5 ÷ 17.5	11.0 ÷ 21.5	{mm}
	Density	ρ	= 2971.5 PD_1_^−0.781^	= 5798.9 PD_2_^−0.918^	{kg/m^3^}
	Bending strength	f_m_	= 1012.9 PD_1_^−1.277^	= 3257 PD_2_^−1.527^	{MPa}
	Bending strength	f_m_’	= 616.84 PD_1_^−0.928^	= 1250.8 PD_2_^−1.059^	{MPa}
	Compressive strength	f_c,0_	= 237.89 PD_1_^−0.742^	= 524.04 PD_2_^−0.927^	{MPa}
	Modulus of elasticity	E	= 52 PD_1_^−0.682^	= 98.638 PD_2_^−0.822^	{GPa}

## Appendix B

**Table A2:** Obtained correlation functions divided into sorting classes.

				PD_1_ {mm}	PD_2_ {mm}	
Pine	KW	Density	ρ	= −33.22 PD_1_ + 856.82	= −22.303 PD_2_ + 848.31	{kg/m^3^}
		Bending strength	f_m_	= −11.6 PD_1_ + 199.02	= −7.782 PD_2_ + 195.96	{MPa}
		Modulus of elasticity	E	= −1.3405 PD_1_ + 27.239	= −0.8266 PD_2_ + 25.788	{GPa}
	KS	Density	ρ	= −41.832 PD_1_ + 963.88	= −32.342 PD_2_ + 1013.8	{kg/m^3^}
		Bending strength	f_m_	= −9.8796 PD_1_ + 179.12	= −7.5332 PD_2_ + 189.14	{MPa}
		Modulus of elasticity	E	= −1.3594 PD_1_ + 27.345	= −1.0637 PD_2_ + 29.18	{GPa}
	KG	Density	ρ	= −22.26 PD_1_ + 720.35 *	= −22.472 PD_2_ + 834.5	{kg/m^3^}
		Bending strength	f_m_	= −3.6176 PD_1_ + 85.399 *	= −3.179 PD_2_ + 96.23 *	{MPa}
		Modulus of elasticity	E	= −0.455 PD_1_ + 14.756 *	= −0.446 PD_2_ + 16.872 *	{GPa}
Spruce	KW	Density	ρ	= −20.593 PD_1_ + 667.71	= −20.926 PD_2_ + 784.76	{kg/m^3^}
		Bending strength	f_m_	= −0.4964 PD_1_ + 52.812	= −0.6619 PD_2_ + 58.424	{MPa}
		Modulus of elasticity	E	= −0.7766 PD_1_ + 19.982	= −0.6322 PD_2_ + 21.616	{GPa}
	KS	Density	ρ	= −29.083 PD_1_ + 782.15	= −21.57 PD_2_ + 806.74	{kg/m^3^}
		Bending strength	f_m_	= −3.1565 PD_1_ + 77.495	= −2.7742 PD_2_ + 87.85	{MPa}
		Modulus of elasticity	E	= −0.7221 PD_1_ + 18.942	= −0.502 PD_2_ + 18.958	{GPa}
	KG	Density	ρ	= −20.291 PD_1_ + 673.12	= −17.348 PD_2_ + 726.35	{kg/m^3^}
		Bending strength	f_m_	= −1.6089 PD_1_ + 54.343 *	= −1.5538 PD_2_ + 61.741 *	{MPa}
		Modulus of elasticity	E	= −0.7534 PD_1_ + 18.753	= −0.6786 PD_2_ + 21.344	{GPa}
Fir	KW	Density	ρ	= −36.286 PD_1_ + 857.75	= −30.493 PD_2_ + 947.62	{kg/m^3^}
		Bending strength	f_m_	= −2.133 PD_1_ + 71.234	= −2.0624 PD_2_ + 80.667	{MPa}
		Modulus of elasticity	E	= −0.4017 PD_1_ + 14.267	= −0.4148 PD_2_ + 16.449	{GPa}
	KS	Density	ρ	= −49.538 PD_1_ + 1013.4	= −43.799 PD_2_ + 1155.4	{kg/m^3^}
		Bending strength	f_m_	= −6.8475 PD_1_ + 124.72	= −6.1616 PD_2_ + 146.07	{MPa}
		Modulus of elasticity	E	= −0.4654 PD_1_ + 14.923	= −0.5068 PD_2_ + 17.783	{GPa}
	KG	Density	ρ	= −10.538 PD_1_ + 540.5	= −11.415 PD_2_ + 603.89 *	{kg/m^3^}
		Bending strength	f_m_	= −2.3566 PD_1_ + 65.605 *	= −2.314 PD_2_ + 76.06 *	{MPa}
		Modulus of elasticity	E	= −0.4455 PD_1_ + 12.809 *	= −0.5873 PD_2_ + 17.121 *	{GPa}

* *p*-value > 0.05—no grounds for rejecting the null hypothesis.

## Appendix C

**Table A3:** Obtained correlation functions transformed to functions corresponding to the 5% safety quantile.

			PD_1_ {mm}	PD_2_ {mm}	
Pine	Scope		7.5 ÷ 15.5	11.0 ÷ 21.0	{mm}
	Density	ρ_5%_	= −34.6·PD_1_ + 825	= −26.3·PD_2_ + 840	{kg/m^3^}
	Bending strength	f_m5%_	= −10.1 PD_1_ + 155	= −7.5·PD_2_ + 160	{MPa}
	Bending strength	f_m_’_5%_	= −6.8 PD_1_ + 128	= −5.0·PD_2_ + 133	{MPa}
	Compressive strength	f_c,05%_	= −4.4 PD_1_ + 80	= −3.3·PD_2_ + 84	{MPa}
	Modulus of elasticity	E_5%_	= −1.35 PD_1_ + 23.3	= −0.9·PD_2_ + 22.5	{GPa}
Spruce	Scope		10.0 ÷ 15.0	15.0 ÷ 21.5	{mm}
	Density	ρ_5%_	= −22.9·PD_1_ +660	= −20.4·PD_2_ + 740	{kg/m^3^}
	Bending strength	f_m5%_	= −2.1·PD_1_ + 56.2	= −2.3·PD_2_ + 71.3	{MPa}
	Bending strength	f_m_’_5%_	= −4.9·PD_1_ + 114.1	= −4.2·PD_2_ + 128.5	{MPa}
	Compressive strength	f_c,05%_	= −2.4·PD_1_ + 60.5	= −2.3·PD_2_ + 70.8	{MPa}
	Modulus of elasticity	E_5%_	= −0.95·PD_1_ +19.9	= −0.7·PD_2_ + 20.4	{GPa}
Fir	Scope		7.5 ÷ 17.5	11.0 ÷ 21.0	{mm}
	Density	ρ_5%_	= −32.3·PD_1_ + 750	= −31.6·PD_2_ + 902	{kg/m^3^}
	Bending strength	f_m5%_	= −3.8·PD_1_ + 76.4	= −3.6·PD_2_ + 90.3	{MPa}
	Bending strength	f_m_’_5%_	= −5.7·PD_1_ + 110.4	= −5.8·PD_2_ + 140.5	{MPa}
	Compressive strength	f_c,05%_	= −1.6·PD_1_ + 51.3	= −1.7·PD_2_ + 62	{MPa}
	Modulus of elasticity	E_5%_	= −0.44·PD_1_ + 12	= −0.48·PD_2_ + 14.6	{GPa}
Species collectively	Scope		7.5 ÷ 17.5	11.0 ÷ 21.5	{mm}
	Density	ρ_5%_	= 2971 PD_1_^−0.85^	= 5799 PD_2_^−0.97^	{kg/m^3^}
	Bending strength	f_m5%_	= 1013 PD_1_^−1.43^	= 3257 PD_2_^−1.65^	{MPa}
	Bending strength	f_m_’_5%_	= 616 PD_1_^−1.03^	= 1250 PD_2_^−1.15^	{MPa}
	Compressive strength	f_c,05%_	= 237 PD_1_^−0.85^	= 524 PD_2_^−1.02^	{MPa}
	Modulus of elasticity	E_5%_	= 52 PD_1_^−0.81^	= 98.7 PD_2_^−0.91^	{GPa}
